# Ultrasound-guided identification of the cricothyroid membrane in a patient with a difficult airway: a case report

**DOI:** 10.1186/s12873-018-0156-7

**Published:** 2018-02-08

**Authors:** Hiromu Okano, Kohji Uzawa, Kunitaro Watanabe, Akira Motoyasu, Joho Tokumine, Alan Kawarai Lefor, Tomoko Yorozu

**Affiliations:** 1 Department of Anesthesiology, Kyorin University School of Medicine, Mailing address: 6-20-2 Shinkawa, Mitaka-shi, Tokyo 181-8611 Japan; 2 Department of Surgery, Jichi Medical University, Mailing address: 3311-1 Yakushiji, Shimotsuke-shi, Tochigi-ken 329-0498 Japan

**Keywords:** Cricothyroid membrane, Cricothyroidotomy, Ultrasound, Difficult airway

## Abstract

**Background:**

Surgical cricothyroidotomy is considered to be the last resort for management of the difficult airway. A major point for a successful surgical cricothyroidotomy is to identify the location of the cricothyroid membrane.

**Case presentation:**

We encountered a patient with progressive respiratory distress who was anticipated to have a difficult airway due to a large neck abscess. We prepared for both awake intubation and surgical cricothyroidotomy. The cricothyroid membrane could not be identified by palpation, but was readily identified using ultrasound.

**Conclusion:**

Ultrasound-guided identification of the cricothyroid membrane may be useful in a patient with a difficult airway due to neck swelling.

## Background

Appropriate treatment of progressive respiratory distress in a patient with a difficult airway is a challenge in emergency care. Decision making must be immediate, with the central question being whether to perform cricothyroidotomy or perform an awake intubation [1]. We encountered a patient with progressive respiratory distress who was considered to be at high risk of a difficult airway due to a large neck abscess. We chose awake intubation and tried simultaneously to identify the cricothyroid membrane. The cricothyroid membrane was not palpable, but was identified promptly using ultrasound.

Emergent control of the airway with awake intubation has no guarantee of success. For patient safety, ultrasound-guided identification of the cricothyroid membrane may be important for smooth surgical cricothyroidotomy if awake intubation fails.

## Case presentation

**Fig. 1:**
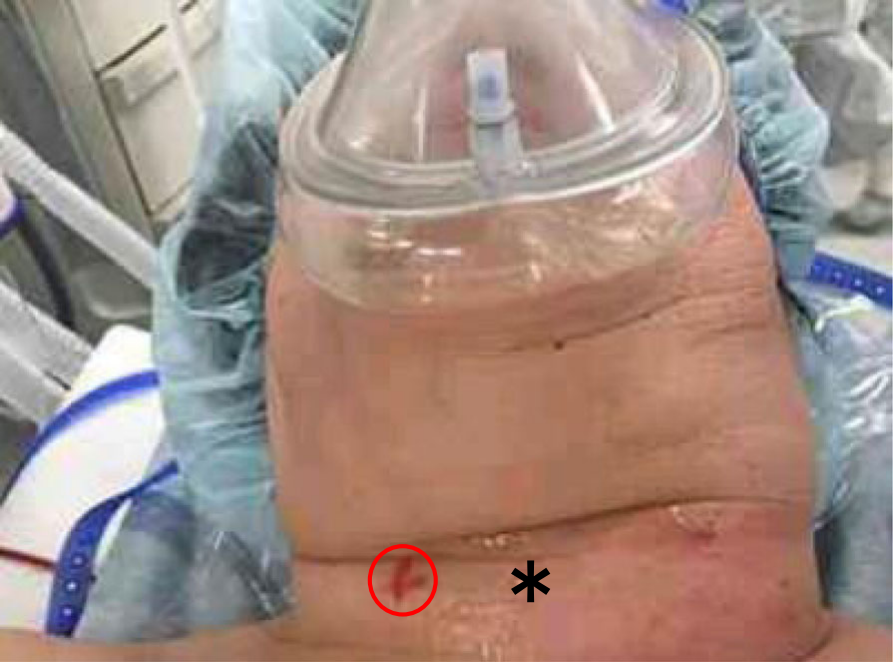
The patient’s neck The asterisk indicates the area palpated by the surgeon to find the cricothyroid membrane. The red circle shows the cricothyroid membrane identified by ultrasound

A 78-year-old woman (148 cm, 52 kg, BMI 27 kg/m^2^) presented with neck pain and swelling. She has no significant past medical history. Physical examination was consistent with a neck abscess (Fig. 1), and she was admitted to the hospital for treatment. Respiratory distress and orthopnea developed, and progressive airway obstruction was suspected as the SpO_2_ fell from 97 to 90%.

**Fig. 2:**
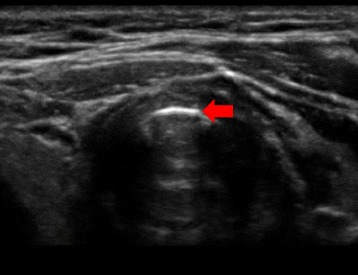
Ultrasound-guided identification of the cricothyroid membrane This is a cross-sectional view of the cervical ultrasound image. The red arrow shows the cricothyroid membrane. The cricothyroid membrane could not be identified by palpation, but was identified on ultrasound

**Fig. 3:**
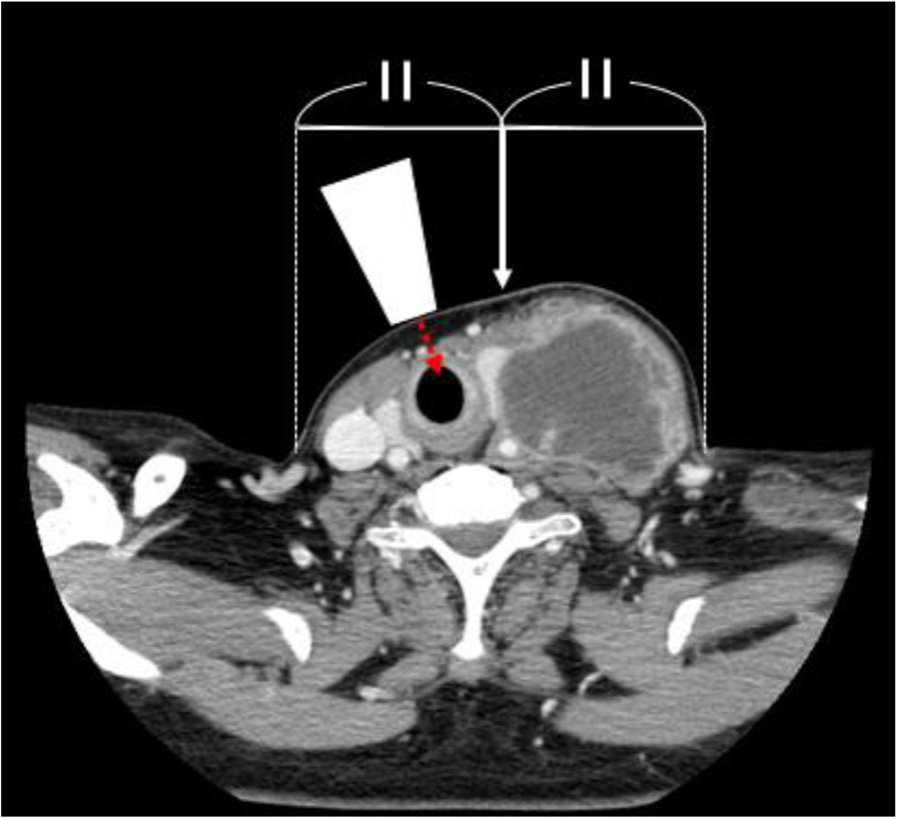
Cervical computed tomography The white arrow indicates the apparent center of the neck. The true center (sagittal line) of the neck is present toward the right side. The ultrasound probe (white trapezoid) is placed perpendicularly to the skin and the ultrasound beam (red dashed arrow) directed to the cricothyroid membrane. The trachea is deviated to the right and rotated to the right

Emergent surgical drainage was planned. Airway management was discussed preoperatively by the surgeon and anesthesiologists and the decision made to perform awake intubation. According to the difficult airway algorithm for the anticipated difficult airway, we prepared the “double set-up preparation for immediate cricotyrotomy” [1]. The anesthesiologists prepared for awake intubation, and the surgeon prepared for cricothyroidotomy. However, the surgeon could not identify the cricothyroid membrane by palpation. The anesthesiologist readily identified the cricothyroid membrane using ultrasound (Fig. 2). The cricothyroid membrane was located more laterally than expected, explaining the difficulty in identifying this structure (Fig. 3).

We performed glossopharyngeal nerve block with topical anesthesia using 8% lidocaine spray, and then sprayed the vocal cords under endoscopic control. Fiberoptic intubation was successfully performed trans-orally without complications. The neck abscess was drained operatively. The patient was treated as an inpatient with antibiotics, and discharged on postoperative day 23, without complications.

## Discussion and conclusions

A “double setup airway intervention” is recommended for the management of patients anticipated to have a difficult airway [1]. The double setup airway intervention means preparing simultaneously for both awake intubation and surgical cricothyroidotomy. A major point in the preparation for a surgical cricothyroidotomy is to identify and mark the location of the cricothyroid membrane. Identification of the precise location of the cricothyroid membrane facilitates a rapid and smooth cricothyroidotomy.

In this patient, the surgeon could not identify the cricothyroid membrane by palpation, but ultrasound easily located this anatomical landmark. The surgeon commented that the cricothyroid membrane in this patient was located more laterally than expected. There were several factors that may have led to difficulty identifying the cricothyroid membrane.

1. The patient’s neck was swollen and protruding to the left side, which caused an illusion that the center of the neck is sagittal. However, the sagittal line was present toward the right side rather than in the center.
2. The trachea and the cricothyroid membrane should be present on the exact sagittal line. However, the trachea and the cricothyroid membrane were deviated to the right side due to the large neck mass.
3. The cricothyroid membrane should be present facing anteriorly. However, the trachea and the cricothyroid membrane were rotated to the opposite side, against the neck mass. Therefore, the front of the cricothyroid membrane facing the skin was more to the right side.

Even after looking at computed tomography scan images, the location of the cricothyroid membrane was not obvious on physical examination. Ultrasound-guided identification of the cricothyroid membrane may be useful in a patient with a difficult airway due to neck swelling. Anatomical identification with palpation has low accuracy [2], especially in female^3,^ [3] and obese [4] patients. The use of ultrasound has been reported to be useful to identify the cricothyroid membrane [5–7]. However, the clinical efficacy of ultrasound guidance to identify the cricothyroid membrane is unclear.

The superior thyroid artery and anterior jugular vein are lateral to the cricothyroid membrane [8]. This is the reason why the initial incision for a cricothyroidotomy is vertical. Fatal airway hemorrhage due to an injured branch of the superior thyroid artery during cricothyroidotomy was reported [9]. Identifying the cricothyroid membrane accurately is the key to performing a safe and successful cricothyroidotomy.

We routinely use transverse scanning to identify the cricothyroid membrane, which is the so-called “TACA method” [6]. The letter “T” refers to the thyroid cartilage, which look triangular in shape on the ultrasound transverse view. The letter T also means “triangle”. The letter “A” indicates air-line, which is the cricothyroid membrane. The letter “C” means the cricoid cartilage, which is hypoechoic, and looks like a black letter “C”. The method involves scanning at the superior thyroid notch then caudally until one can identify the cricothyroid membrane, then pass it and return to verify it.

We usually use the superior thyroid notch as a landmark when starting transverse scanning. It can be easily identified as a prominence in the upper neck, especially in males, and is known as the “Adam’s apple”. Unfortunately, this prominence is relatively unclear in females. However, the thyroid cartilage is easily identified.

In this report, the anesthesiologist did not use the usual transverse scan technique. He could not identify the superior thyroid notch due to the patient’s swollen neck. He started the scanning from the lower neck which looked anatomically normal. At first, he found the trachea which is the “C” portion of the TACA method, then scanned cranially to identify the cricothyroid membrane. We cannot recommend one standard method to identify the structures in patients with a difficult airway, but we strongly recommend trying to identify the thyroid cartilage or cricoid cartilage. The cricothyroid membrane should be present between them.

Point-of-care ultrasound is rapidly becoming an important tool in a new era. The use of ultrasound for evaluation of the airway and identification of the cricothyroid membrane may become a basic tool and mandatory skill for the practice of anesthesia in the near future. Recent progress in ultrasound examinations for airway management may reveal its efficacy, but may also reveal its limitations. This may include patients with subcutaneous emphysema or gas gangrene.

This case report suggests the possible efficacy of ultrasound identification in airway management in patients with an anticipated difficult airway. However, ultrasound use in patients with an unanticipated difficult airway might be limited because of the time needed for equipment setup. If the ultrasound machine were present for all patients, and start-up of the machine is instant and if the operator had the skills to identify the cricothyroid membrane immediately, this skill may be useful in patients with an unanticipated difficult airway. The most important factor for successful cricothyroidotomy is thought to be correct identification of the cricothyroid membrane [5]. Cricothyroidotomy is thought of as the procedure of last resort in case of “cannot intubate, cannot oxygenate”. However, even in these patients, contraindications to cricothyroidotomy may exist, such as a subglottic tumor [10].

Using ultrasound, the cricothyroid membrane in a patient with neck swelling was readily identified. Determining the clinical efficacy of ultrasound identification of the cricothyroid membrane for anticipated difficult airway management requires further study.
